# Production of IgG2 Antibodies to Pneumococcal Polysaccharides After Vaccination of Treated HIV Patients May Be Augmented by IL-7Rα Signaling in ICOS^+^ Circulating T Follicular-Helper Cells

**DOI:** 10.3389/fimmu.2019.00839

**Published:** 2019-04-24

**Authors:** Laila N. Abudulai, Sonia Fernandez, Karli Corscadden, Lea-Ann Kirkham, Michael Hunter, Jeffrey J. Post, Martyn A. French

**Affiliations:** ^1^School of Biomedical Sciences, The University of Western Australia, Perth, WA, Australia; ^2^Centre for Microscopy, Characterisation and Analysis, The University of Western Australia, Perth, WA, Australia; ^3^Wesfarmers Centre for Vaccine and Infectious Disease Research, Telethon Kids Institute, The University of Western Australia, Perth, WA, Australia; ^4^Department of Infectious Diseases, Royal Victoria Hospital, Belfast, United Kingdom; ^5^Department of Infectious Diseases, Prince of Wales Hospital, Sydney, NSW, Australia; ^6^Prince of Wales Clinical School, University of New South Wales, Sydney, NSW, Australia; ^7^UWA Medical School, The University of Western Australia, Perth, WA, Australia

**Keywords:** T follicular cells, IgG2 antibodies, pneumococcal 23-polyvalent vaccine, HIV infection, IL-7 receptor

## Abstract

Greater understanding of factors influencing the maturation of antibody responses against pneumococcal polysaccharides (PcPs) may improve pneumococcal vaccination strategies. Although PcPs are type 2 T cell-independent antigens thought not to induce follicular immune responses, we have previously shown that IgG2 antibody responses against antigens in the 23-valent unconjugated PcP vaccine (PPV23) are associated with expansion of ICOS^+^ circulating T follicular helper (cT_FH_) cells in HIV seronegative subjects but not HIV patients. As IL-7Rα signaling in CD4^+^ T cells may affect T_FH_ cell function and is adversely affected by HIV-1 infection, we have examined the relationship of IL-7Rα expression on ICOS^+^ cT_FH_ cells with PcP-specific IgG2 antibody responses. PPV23 vaccination was undertaken in HIV patients receiving antiretroviral therapy (*n* = 25) and HIV seronegative subjects (*n* = 20). IL-7Rα expression on ICOS^+^ and ICOS^−^ cT_FH_ cells was assessed at day(D) 0, 7, and 28. Fold increase between D0 and D28 in serum IgG1 and IgG2 antibodies to PcP serotypes 4, 6B, 9V, and 14 and the frequency of IgG1^+^ and IgG2^+^ antibody secreting cells (ASCs) at D7 were also assessed. Decline in IL-7Rα expression on ICOS^+^ cT_FH_ cells between D0 and D7 occurred in 75% of HIV seronegative subjects and 60% of HIV patients (Group A), with changes in IL-7Rα expression being more pronounced in HIV patients. Group A patients exhibited abnormally high IL-7Rα expression pre-vaccination, an association of serum IgG2, but not IgG1, antibody responses with a decline of IL-7Rα expression on ICOS^+^ cT_FH_ cells between D0 and D7, and an association of higher IgG2^+^ ASCs with lower IL-7Rα expression on ICOS^+^ cT_FH_ cells at D7. As decline of IL-7Rα expression on CD4^+^ T cells is an indicator of IL-7Rα signaling, our findings suggest that utilization of IL-7 by cT_FH_ cells affects production of IgG2 antibodies to PPV23 antigens in some HIV patients.

## Introduction

Although the immunogenicity of pneumococcal vaccines is increased by protein conjugation of pneumococcal polysaccharides (PcPs), leading to recruitment of T cell “help” (1), strategies for enhancing IgG antibody production and the generation of memory B cells (MBCs) are still needed. This is particularly so for people at greatest risk of pneumococcal disease, including patients with human immunodeficiency virus (HIV) infection treated with antiretroviral therapy (ART). While the 13-valent conjugated pneumococcal vaccine (PCV13) is more effective than the 23-valent unconjugated pneumococcal vaccine (PPV23) in HIV patients, IgG antibody responses are lower than in HIV seronegative subjects (2, 3). Furthermore, PCV13 does not provide the breadth of pneumococcal serotype coverage that PPV23 does and current recommendations are to vaccinate with PCV13 followed by PPV23 (4).

Pneumococcal polysaccharides are type 2 T-independent (TI-2) antigens, which can induce antibodies without T cell help (5). T cell-independent antibody responses consist predominantly of IgM antibodies and, furthermore, vaccination with PcPs induces predominantly IgM^+^ memory B cells (MBCs) (6). It is generally considered that these characteristics reflect production of PcP-specific antibodies and MBCs at extrafollicular sites. However, in addition to IgM antibodies, IgG antibodies and particularly those of the IgG2 subclass are an important component of opsonophagocytic antibody responses against pneumococcal polysaccharides (7). Indeed, IgG2 deficiency is associated with an increased susceptibility to infections of the respiratory tract by encapsulated bacteria, including pneumococci (8, 9).

Plasma cells producing IgG2 antibodies are derived from IgG^+^ MBCs (10, 11) and analyses of VDJ gene mutation levels and the degree of somatic hypermutation (SHM) within immunoglobulin genes of these cells suggest that they differentiate in germinal center (GC) reactions, particularly secondary GC reactions (10, 12). IgM^+^ MBCs also express transcripts of *IGHG2* and surface IgG2 when activated by neutrophils, but it is unclear if they differentiate directly into IgG2^+^ antibody secreting cells (ASCs) or following entry into GCs (13). We have previously shown that vaccination with PPV23 is associated with increased frequencies of circulating follicular helper T (cT_FH_) cells expressing inducible co-stimulator (ICOS) (ICOS^+^ cT_FH_ cells) (14). We have also shown that the frequencies of ICOS^+^ cT_FH_ cells correlated with IgG1^+^ and particularly IgG2^+^ ASCs at D7 post-vaccination in HIV seronegative subjects but not HIV patients (14). As ICOS^+^ cT_FH_ cells represent the circulating counterpart of activated follicular helper T (T_FH_) cells (15, 16), which are critical for GC reactions and may affect vaccine-induced antibody responses (17), we have proposed that GC reactions might contribute to the maturation of PcP vaccine-induced antibody responses and are impaired in patients with treated HIV-1 infection because of lymph node fibrosis (14).

In humans, terminal differentiation of T_FH_ cells is marked by loss of interleukin-7 receptor alpha (IL-7Rα; CD127) expression (18). As IL-7Rα expression on murine T_FH_ cells may influence vaccine-induced antibody responses (19, 20), it is possible that IL-7 binding to IL-7Rα on ICOS^+^ cT_FH_ cells may contribute to the regulation of IgG2 antibody production after PPV23 vaccination in humans. The receptor for IL-7 is a heterodimer of the α subunit (IL-7Rα) and the cytokine receptor common γ chain (γc). On IL-7 binding, heterodimerization of IL-7Rα and γc (CD132) activates the Jak/STAT signaling pathway (21) and downregulation of IL-7Rα expression by decreasing its gene expression (22). IL-7Rα is highly expressed on naive and central memory T-cells and downregulated when activated by antigens (23). The frequency of cT_FH_ cells (CD4^+^CD45RO^+^CXCR5^+^) expressing IL-7Rα and the level of receptor expression are similar in ART-treated HIV patients and HIV seronegative subjects (24). However, HIV patients may exhibit defects of IL-7Rα signaling in CD4^+^ T cells that are not related to the amount of receptor expression (25) and some ART-treated HIV patients continue to exhibit decreased IL-7Rα signaling in CD4^+^ T cells (26).

To investigate the relationship between cT_FH_ cell function and PcP-specific IgG2 antibody responses, we have examined IL-7Rα expression on ICOS^+^ cT_FH_ cells before and after PPV23 vaccination and related findings to the increase in frequency of ICOS^+^ cT_FH_ cells, fold-increase in serum IgG1 and IgG2 PcP antibody levels and IgG1^+^ and IgG2^+^ PcP-specific ASCs after vaccination of ART-treated HIV patients and HIV seronegative subjects. We report the novel finding that production of PcP-specific IgG2 antibodies in ART-treated HIV patients was associated with abnormally high IL-7Rα expression on ICOS^+^ cT_FH_ cells at D0 and a decline of IL-7Rα expression on ICOS^+^ cT_FH_ cells between D0 and D7.

## Methods

### Study Participants

HIV patients receiving ART (*n* = 25) and age-matched HIV-seronegative subjects (*n* = 20) were recruited to the study if they had not previously received a pneumococcal vaccine. All participants were >18 years old and HIV patients were attending clinics in Perth or Sydney. The HIV patients were subsequently separated into two groups based on whether IL-7Rα expression on ICOS^+^ cT_FH_ cells at day 7 was decreased (Group A) or not (Group B) (see later). Demographic and clinical data are described in Supplementary Table 1. Study participants were vaccinated with 0.5 mL PPV23 (Pneumovax^TM^, Merck, Sharp, and Dohme, Whitehouse Station, NJ, USA). Informed written consent was obtained from all participants and the study was approved by the Human Research Ethics Committees of Royal Perth Hospital, Perth, Australia (2011/027), the University of Western Australia, Perth, Australia (RA/4/1/4871) and Prince of Wales Hospital, Sydney, Australia (11/020).

### Processing of Blood Samples

Blood was collected before vaccination (D0), and 1 week (range 7–8 days, D7) and 4 weeks (range 28–37 days, D28) after vaccination. Serum and PBMC isolated by density separation were cryopreserved until analysis. Whole blood CD4^+^ T cell counts were assayed in laboratories accredited by the National Association of Testing Authorities, Australia.

### Expression of IL-7Rα on ICOS^+^ and ICOS^−^ cT_FH_ Cells

**Figure 1:**
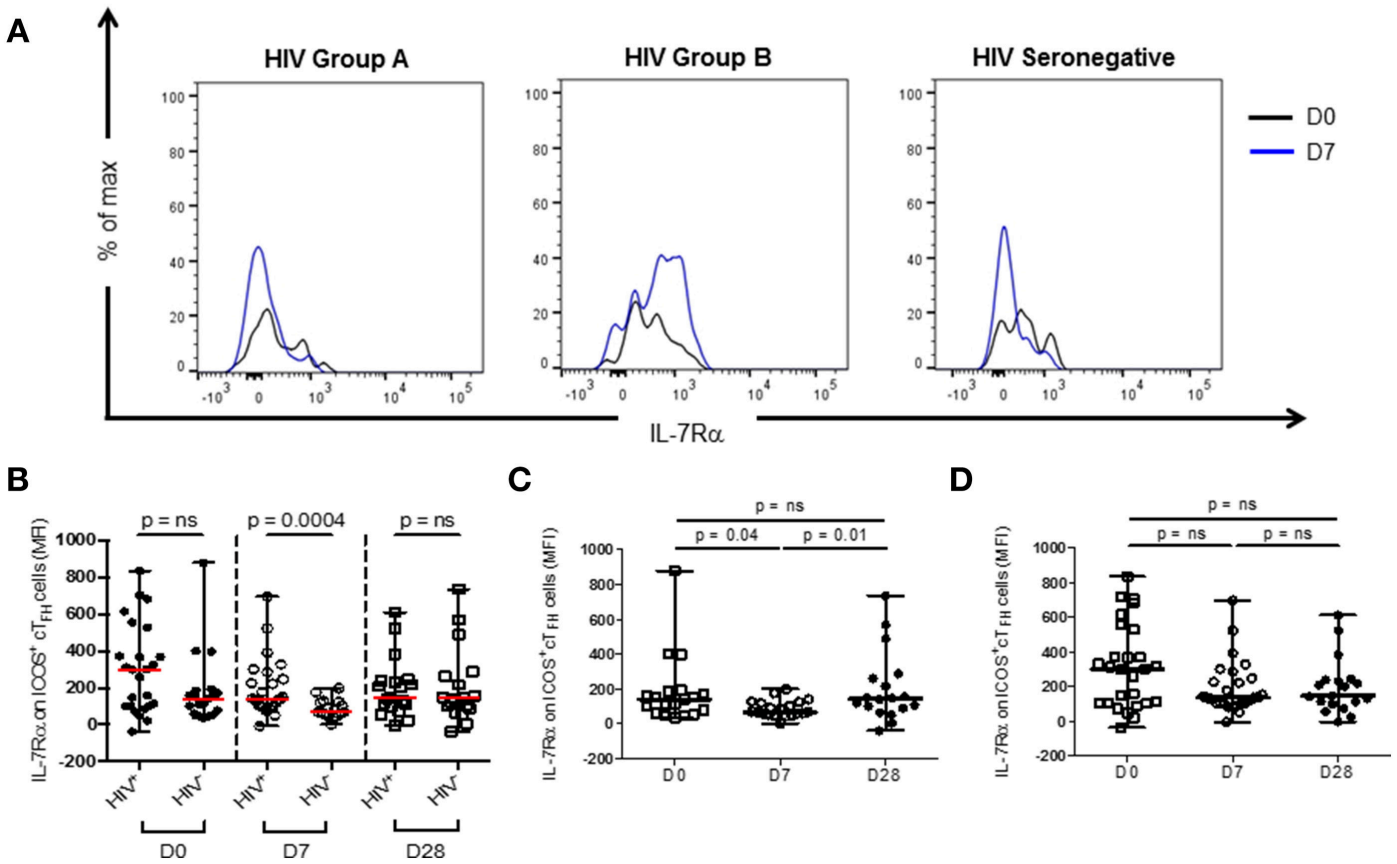
IL-7Rα expression on ICOS^+^ cT_FH_ cells at D0, D7, and D28 of vaccination with PPV23 in HIV patients and HIV seronegative subjects. **(A)** Representative histogram plots demonstrating IL-7Rα expression at D0 and D7 in a treated HIV Group A (left panel) and HIV Group B patient (middle panel) and an HIV seronegative subject (right panel). **(B)** Comparison of IL-7Rα expression on ICOS^+^ cT_FH_ of HIV patients and HIV seronegative subjects. **(C)** IL-7Rα expression on ICOS^+^ cT_FH_ cells of HIV seronegative subjects and **(D)** IL-7Rα expression on ICOS^+^ cT_FH_ cells of ART-treated HIV patients. Horizontal lines represent medians with ranges. Differences between groups were assessed using Mann-Whitney test. Differences between time-points were assessed using Wilcoxon signed-rank test. n.s., not significant and *p* < 0.05, significant.

Thawed PBMC were stained with CD3-V450 (clone UCHT1), CD4-FITC (clone RPA-T4), CD27-BV510 (L128), CXCR5-BV421 (RF8B2), ICOS-PerCp-Cy5.5 (DX29), PD-1-APC (MIH4), CD45RA-APC-H7 (HI100), and IL-7Rα-PerCp-Cy5.5 (HIL-7R-M21) (BD Biosciences, San Jose, CA) for 20 min, washed and re-suspended in PBS with 1% BSA. Analyses were performed using a FACS Canto II cytometer (BD Biosciences). A minimum of 200,000 CD4 events was acquired for each sample. Data was visualized using FlowJo software (V10, Tree Star, Ashland, OR). The gating strategy used to identify ICOS^+^ cT_FH_ cell populations has been previously described (14). IL-7Rα expression was assessed as the median fluorescence intensity (MFI) of expression on ICOS^+^ cT_FH_ cells (Figure 1A).

### Assays for PcP-Specific IgG1^+^ and IgG2^+^ ASCs and Serum Antibodies

ELISpot assays to enumerate IgG1^+^ and IgG2^+^ ASCs in cryopreserved PBMC, and ELISAs to quantitate serum IgG1 and IgG2 antibodies specific for PcP serotypes 4, 6B, 9V, and 14, were undertaken as previously described (14).

### Statistical Analyses

Differences in baseline characteristics between groups were tested using Mann Whitney U-test or Fisher's exact test as appropriate. Correlation coefficients between variables were evaluated by the non-parametric Spearman's rank correlation test. Non-parametric tests (Wilcoxon signed rank test for within group and Mann Whitney for between groups) were performed for all other comparisons. Statistical analyses were performed using Prism Version 5.04 software (GraphPad). For all tests, *p* < 0.05 was considered significant.

## Results

### IL-7Rα Expression on ICOS^+^ cT_FH_ Cells Declined Between D0 and D7 of Vaccination in a Proportion of HIV Patients and HIV Seronegative Subjects

Expression levels (MFI) of IL-7Rα were examined on ICOS^+^ cT_FH_ cells of HIV seronegative subjects and ART-treated HIV patients at D0, D7, and D28 after vaccination with PPV23 (Figure 1). Expression of IL-7Rα on ICOS^+^ cT_FH_ cells before vaccination in HIV patients was not different from HIV seronegative subjects (Figure 1B). In contrast, IL-7Rα expression on ICOS^+^ cT_FH_ cells was higher and much more variable in HIV patients than HIV seronegative subjects at D7 (Figure 1B). There was no difference at D28.

**Figure 2:**
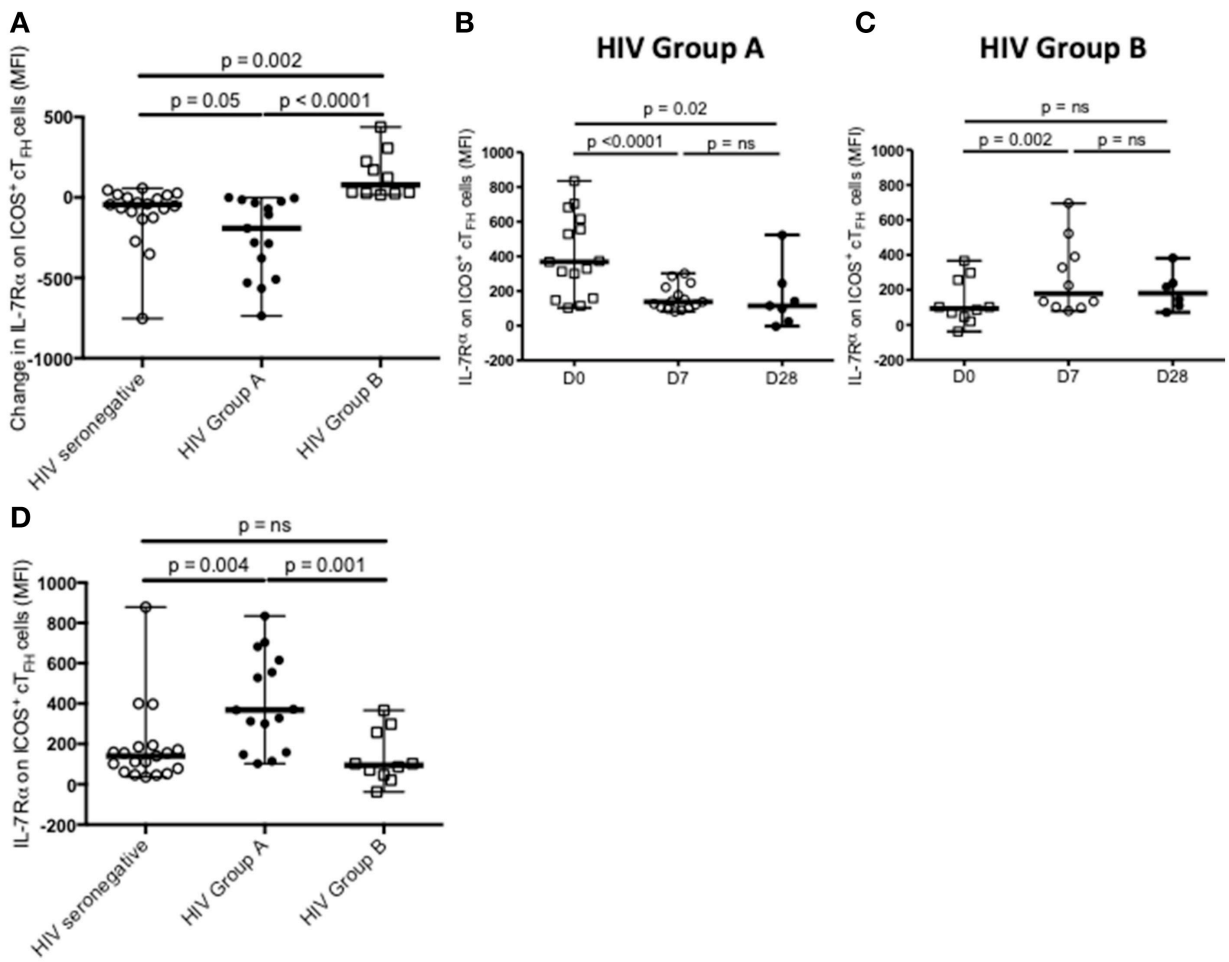
Definition and characterization of Group A and Group B HIV patients. **(A)** Change in IL-7Rα expression on ICOS^+^ cT_FH_ cells from D0 to D7 defined Group A (*n* = 15) and Group B (*n* = 10) HIV patients, which were compared with HIV seronegative subjects (*n* = 20). **(B)** IL-7Rα expression on ICOS^+^ cT_FH_ cells of Group A HIV patients at D0, D7, and D28. **(C)** IL-7Rα expression on ICOS^+^ cT_FH_ cells of Group B HIV patients at D0, D7, and D28. **(D)** Comparison of IL-7Rα expression on ICOS^+^ cT_FH_ cells at D0 in Group A and Group B HIV patients and HIV seronegative subjects. Horizontal lines represent medians with ranges. Differences between time-points were assessed using Wilcoxon signed-rank tests. Differences between groups were assessed using Mann-Whitney test. n.s., not significant and *p* < 0.05, significant.

We next examined change in IL-7Rα expression on ICOS^+^ cT_FH_ cells between D0 and D7. In HIV seronegative subjects, IL-7Rα expression declined between D0 and D7 in the total group (*p* = 0.04, Figure 1C) but only 15 of the 20 subjects (75%) exhibited a decline. In HIV patients, there was not a statistically significant difference in change of IL-7Rα expression on ICOS^+^ cT_FH_ cells between D0 and D7 (Figure 1D) and similar to HIV seronegative subjects, 15 of 25 HIV patients (60%) exhibited a decline in IL-7Rα expression on ICOS^+^ cT_FH_ cells between D0 and D7. However, both decline and increase of IL-7Rα expression were more pronounced in HIV patients compared with HIV seronegative subjects. We therefore examined patients exhibiting a decline (referred to as Group A, *n* = 15) or no decline (Group B, *n* = 10) in IL-7Rα expression separately (Figure 2A). Notably, the pattern of change in IL-7Rα expression in Group A and B patients persisted at day D28 (Figures 2B, C).

**Figure 3:**
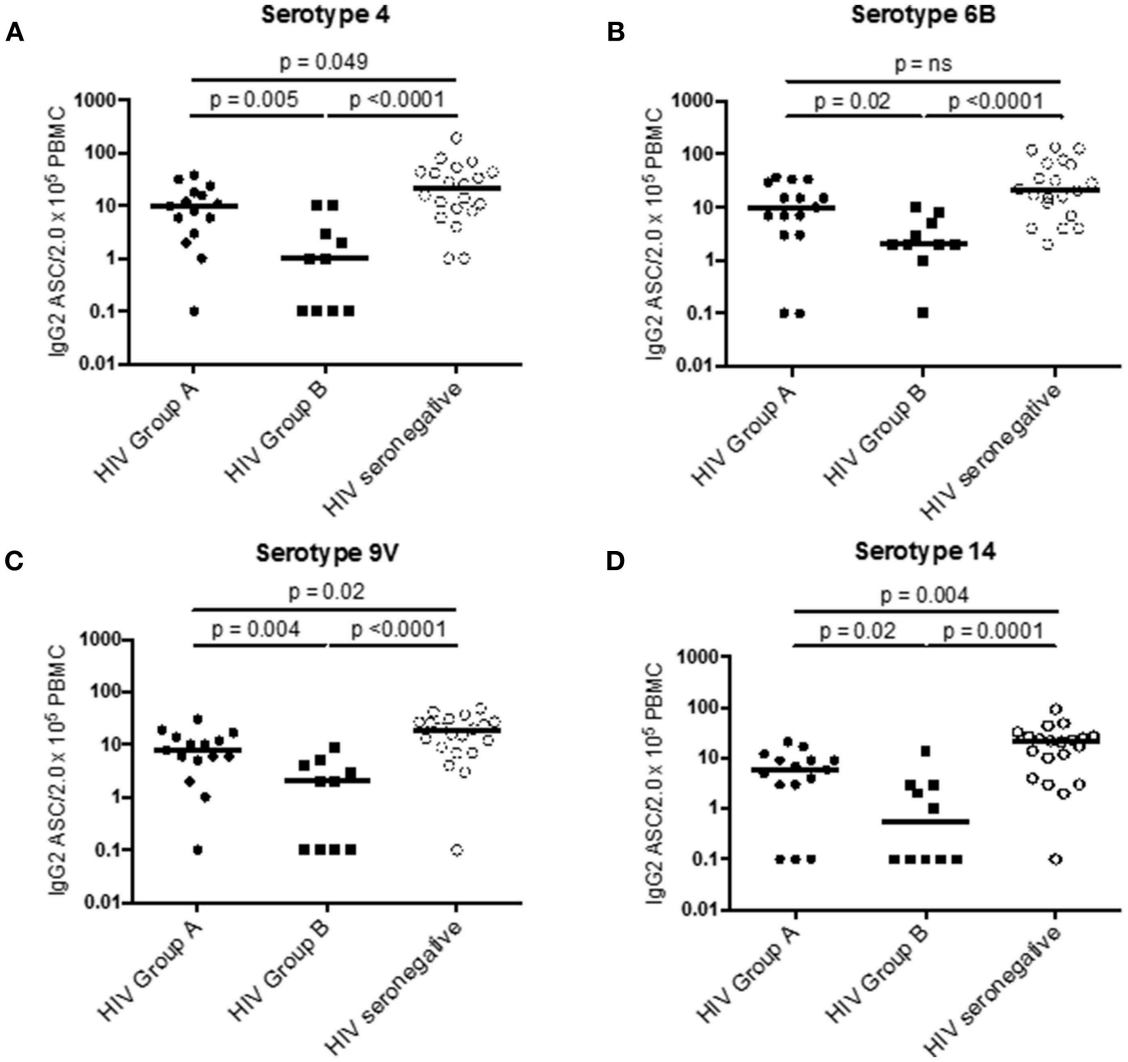
PcP serotype specific IgG2^+^ ASC in Group A HIV patients, Group B HIV patients, and HIV seronegative subjects at D7 post-vaccination with PPV23. **(A)** PcP serotype 4. **(B)** PcP serotype 6B. **(C)** PcP serotype 9V. **(D)** PcP serotype 14. Differences between groups were assessed using Mann-Whitney test. n.s., not significant and *p* < 0.05, significant. IgG2^+^ ASC values of 0 were given a value of 0.1 for analysis of data on a log scale.

To compare the two subgroups of HIV patients, we examined IL-7Rα expression on ICOS^+^ cT_FH_ cells at D0, also examining this in HIV seronegative subjects. ICOS^+^ cT_FH_ cells of Group A HIV patients expressed more IL-7Rα compared to HIV Group B patients and HIV seronegative subjects (*p* = 0.001 and *p* = 0.004, respectively, Figure 2D), who did not differ from each other (Figure 2D). In addition, ASC production after vaccination was compared in the three study groups (Figure 3) and demonstrated that Group A HIV patients exhibited higher IgG2^+^ ASCs for all four PcP serotypes compared with Group B HIV patients, though both groups had lower values than HIV seronegative subjects, as previously reported (14). There were no differences between Group A and Group B HIV patients for IgG1^+^ ASCs at D7 (Supplementary Figure 1).

Interestingly, IL-7Rα expression on ICOS^−^ cT_FH_ cells of HIV seronegative subjects was higher at D28 compared to D0 and D7 (*p* = 0.02 and *p* = 0.01), respectively, whereas there was no change in HIV patients (Supplementary Figure 2).

### Expansion of ICOS^+^ cT_FH_ Cells Between D0 and D7 in HIV Seronegative Subjects Was Associated With Lower IL-7Rα Expression on ICOS^+^ cT_FH_ Cells at D0 or a Decline of IL-7Rα Expression Between D0 and D7

**Figure 4:**
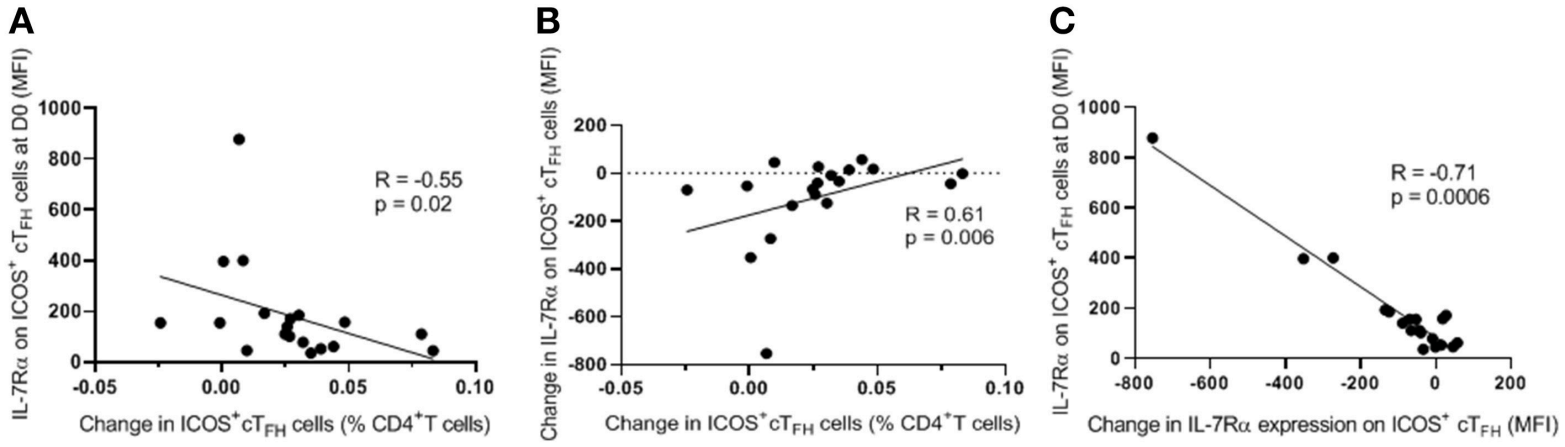
Correlations of change in frequency of ICOS^+^ cT_FH_ cells between D0 and D7 with level of IL-7Rα expression on ICOS^+^ cT_FH_ cells at D0 or change in IL-7Rα expression between D0 and D7. **(A)** Change in frequency of ICOS^+^ cT_FH_ cells between D0 and D7 inversely correlated with the level of IL-7Rα expression on ICOS^+^ cT_FH_ cells at D0. **(B)** Change in frequency of ICOS^+^ cT_FH_ cells between D0 and D7 positively correlated with change in IL-7Rα expression on ICOS^+^ cT_FH_ cells between D0 and D7. **(C)** Change in IL-7Rα expression between D0 and D7 inversely correlated with IL-7Rα expression at D0. Data were analyzed using Spearman's rank correlation test.

To examine functional correlates of IL-7Rα expression on ICOS^+^ cT_FH_ cells at D0 and change in IL-7Rα expression between D0 and D7, we correlated these parameters with the change in frequency of ICOS^+^ cT_FH_ cells between D0 and D7 in HIV seronegative subjects because we have previously shown that PcP-specific IgG2 antibody responses are associated with expansion of ICOS^+^ cT_FH_ cells between D0 and D7 in HIV seronegative subjects but not HIV patients (14). As shown in Figure 4A, change in frequency of ICOS^+^ cT_FH_ cells between D0 and D7 inversely correlated with IL-7Rα expression on ICOS^+^ cT_FH_ cells at D0 (*R* = −0.55, *p* = 0.02; Figure 4A) but positively with change in IL-7Rα expression between D0 and D7 (*R* = 0.61, *p* = 0.006, Figure 4B). Furthermore, change in IL-7Rα expression between D0 and D7 inversely correlated with IL-7Rα expression at D0 (*R* = −0.71, *p* = 0.0006; Figure 4C), indicating that decline in IL-7Rα expression on ICOS^+^ cT_FH_ cells was greatest in subjects with the highest IL-7Rα expression at D0. Thus, expansion of ICOS^+^ cT_FH_ cells after vaccination was associated with lower IL-7Rα expression on ICOS^+^ cT_FH_ cells at D0 or a greater decline in IL-7Rα expression between D0 and D7.

### Decline in IL-7Rα Expression on ICOS^+^ cT_FH_ Cells of Group A Patients Was Associated With Higher Nadir and Current CD4^+^ T-Cell Counts

Current and nadir CD4^+^ T-cell counts did not differ between Group A and Group B HIV patients (*p* = 0.71 and *p* = 0.26, respectively; data not shown). However, in Group A HIV patients only, decline in IL-7Rα expression (D7–D0) on ICOS^+^ cT_FH_ cells negatively correlated with both nadir (*R* = −0.53, *p* = 0.04) and current (*R* = −0.48, *p* = 0.08) CD4^+^ T-cell counts (data not shown). Thus, the greatest decline of IL-7Rα expression was observed in patients with the highest nadir and current CD4^+^ T-cell counts.

### Serum PcP-Specific IgG2 Antibody Responses Were Associated With a Decline in IL-7Rα Expression on ICOS^+^ cT_FH_ Cells in Group A HIV Patients

**Figure 5:**
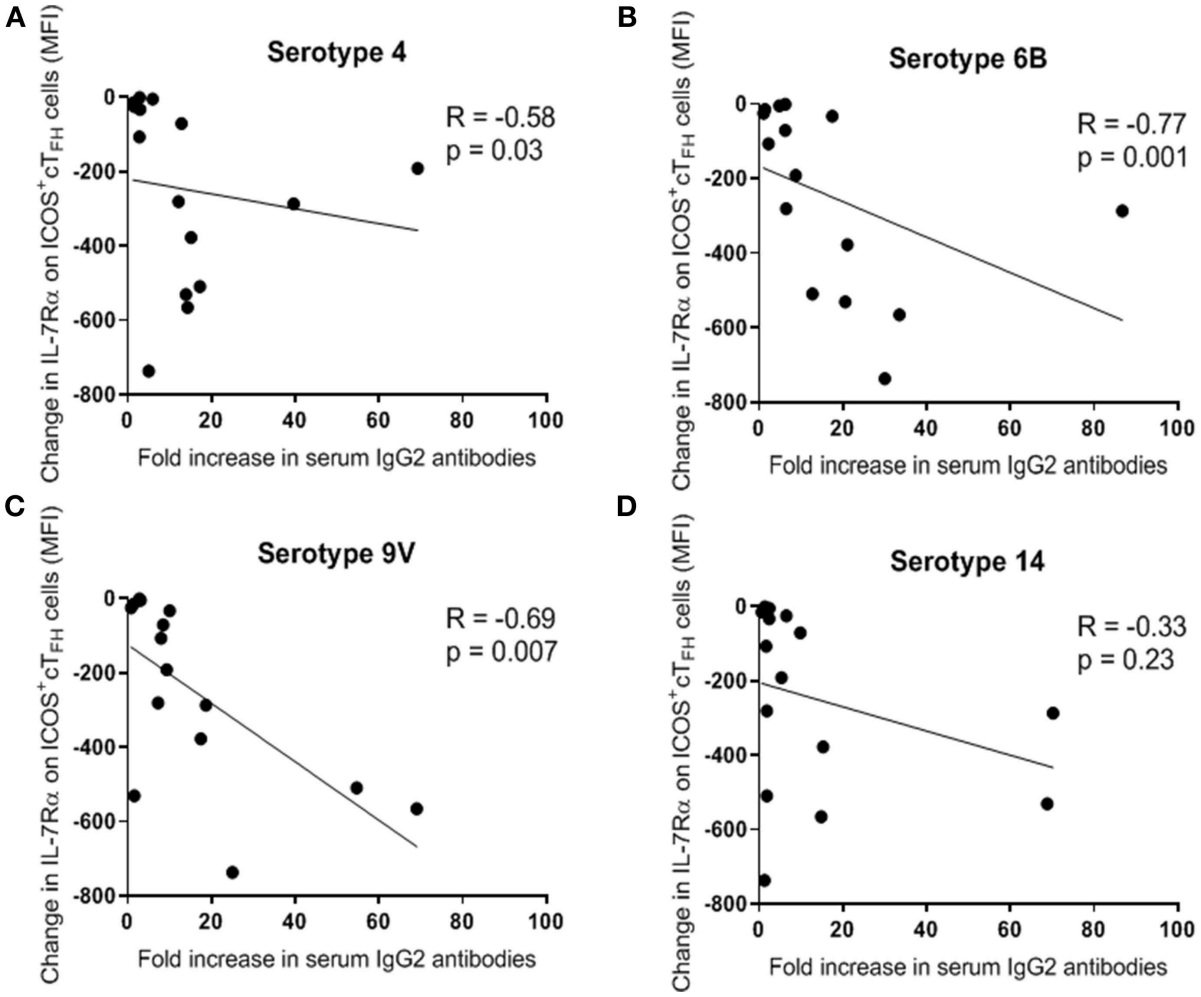
Change in expression of IL-7Rα on ICOS^+^ cT_FH_ cells between D0 and D7 inversely correlated with fold-increase (between D0 and D28) in serum levels of PcP serotype-specific IgG2 antibodies in Group A HIV patients. **(A)** PcP serotype 4. **(B)** PcP serotype 6B. **(C)** PcP serotype 9V. **(D)** PcP serotype 14. Data were analyzed using Spearman's rank correlation test.

We next investigated the relationship between change in IL-7Rα expression on ICOS^+^ cT_FH_ cells between D0 and D7 and fold-change in serum PcP-specific IgG1 and IgG2 antibody levels between D0 and D28 in both groups of HIV patients and HIV seronegative subjects. In Group A HIV patients, fold-increase in IgG2 antibodies to PcP serotypes 4, 6B, and 9V inversely correlated with change in IL-7Rα expression on ICOS^+^ cT_FH_ cells (*R* = −0.58 to 0.77, *p* ≤ 0.03; Figures 5). In contrast, there were no correlations in Group B HIV patients or HIV seronegative subjects. Furthermore, there were no correlations between fold-increase in serum IgG1 PcP-specific antibodies and change in IL-7Rα expression on ICOS^+^ cT_FH_ cells in any group (data not shown).

### Lower IL-7Rα Expression on ICOS^+^ cT_FH_ Cells Was Associated With Higher IgG2^+^ ASC Frequencies at D7 in Group A HIV Patients

**Figure 6:**
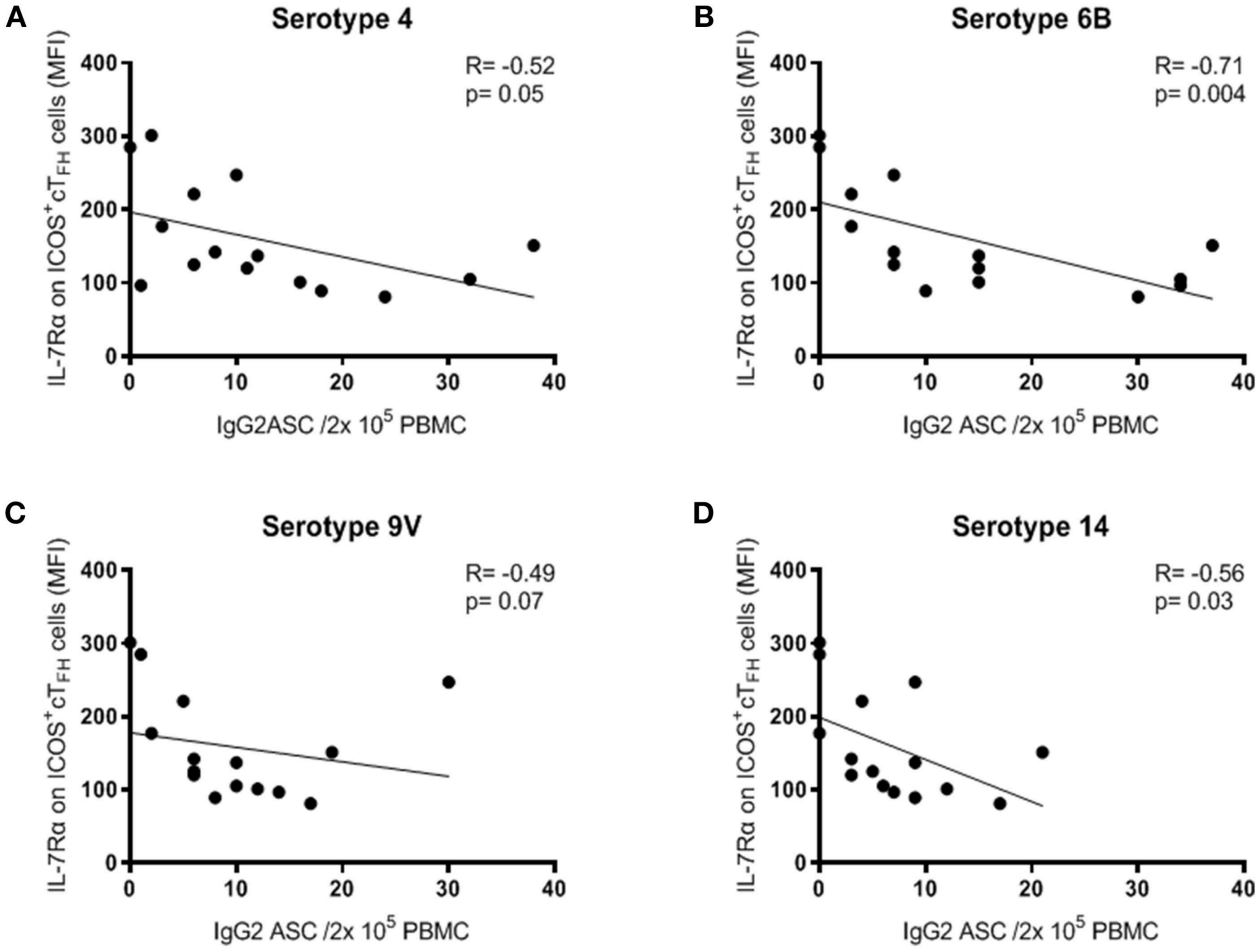
Expression of IL-7Rα on ICOS^+^ cT_FH_ cells at D7 correlated inversely with IgG2^+^ ASC at D7 following PPV23 vaccination in HIV Group A patients. **(A)** PcP serotype 4. **(B)** PcP serotype 6B. **(C)** PcP serotype 9V. **(D)** PcP serotype 14. Data were analyzed using Spearman's rank correlation test.

Further evidence of a relationship between IL-7Rα expression on ICOS^+^ cT_FH_ cells and PcP-specific IgG2 antibody responses was provided by examining ASCs at D7. Change in IL-7Rα expression on ICOS^+^ cT_FH_ cells between D0 and D7 did not correlate with ASCs at D7 in either HIV patient group. However, in Group A HIV patients, the frequency of IgG2^+^ ASCs for all 4 PcPs inversely correlated with IL-7Rα expression on ICOS^+^ cT_FH_ (R ≥ −0.49, p≤0.07; Figure 6). Thus, a higher frequency of IgG2^+^ ASCs was associated with lower expression of IL-7Rα on ICOS^+^ cT_FH_ cells at D7. In contrast, the frequency of IgG2^+^ ASCs did not correlate with IL-7Rα expression on ICOS^+^ cT_FH_ cells at D7 in HIV Group B patients to 3 of 4 PcPs (*R* ≥ −0.21, *p* ≥ 0.09; data not shown) or HIV seronegative subjects (*R* ≤ −0.03, *p* ≥ 0.87; data not shown). Of note, IL-7Rα expression on ICOS^+^ cT_FH_ cells at D7 did not correlate with IgG1^+^ ASCs in any group (data not shown).

## Discussion

We have demonstrated that 75% of HIV seronegative subjects and 60% of ART-treated HIV patients exhibited a decline in IL-7Rα expression on ICOS^+^ cT_FH_ cells, the activated subpopulation of cT_FH_ cells (15, 16), between D0 and D7 of vaccination with PPV23. Decline in IL-7Rα expression, as well as increased IL-7Rα expression, was more pronounced in HIV patients. Amongst HIV patients who exhibited a decline in IL-7Rα expression on ICOS^+^ cT_FH_ cells (Group A), a greater decline in IL-7Rα expression on ICOS^+^ cT_FH_ cells between D0 and D7 was associated with higher serum IgG2 but not IgG1 PcP-specific antibody responses. In contrast, IgG2 antibody responses were not related to change in IL-7Rα expression on ICOS^+^ cT_FH_ cells between D0 and D7 in HIV patients who did not exhibit a decline in IL-7Rα expression on ICOS^+^ cT_FH_ cells (Group B), who were different to Group A HIV patients in several regards. Interestingly, HIV seronegative subjects also did not exhibit an association of decline in IL-7Rα expression on ICOS^+^ cT_FH_ cells between D0 and D7 with IgG2 PcP-specific antibody responses, even though we demonstrated a relationship with expansion of ICOS^+^ cT_FH_ cells between D0 and D7 (Figure 4B). We suggest that this reflected the smaller dynamic range of values for change of IL-7Rα expression on ICOS^+^ cT_FH_ cells, compared with HIV patients (Figure 2A), reducing the power of correlation analyses for antibody responses.

As loss of IL-7Rα expression is indicative of the terminal differentiation of T_FH_ cells (18) and binding of IL-7 to the IL-7R causes downregulation of IL-7Rα expression (22), our interpretation of these findings is that PcP-specific IgG2 antibody responses are associated with expansion of ICOS^+^ cT_FH_ cells (14) that have undergone differentiation and/or utilized IL-7. In support of this proposal, we also demonstrated that expansion of ICOS^+^ cT_FH_ cells after vaccination of HIV seronegative subjects was associated with lower IL-7Rα expression on ICOS^+^ cT_FH_ cells at D0 or a greater decline in IL-7Rα expression between D0 and D7 (Figure 4). Indeed, these findings might suggest that subgroups of responder phenotype also exist amongst HIV seronegative subjects. However, a robust analysis of this could not be undertaken because of an insufficient number of subjects.

In agreement with Chiodi et al. (24), whose cT_FH_ cell immunophenotyping panel did not differentiate between activated and memory cT_FH_ cells, we found that IL-7Rα expression on ICOS^+^ cT_FH_ cells was not significantly different in HIV seronegative subjects and ART-treated HIV patients at D0 or D28 of vaccination. However, at D7, IL-7Rα expression on ICOS^+^ cT_FH_ cells was higher and more variable in HIV patients compared with HIV seronegative subjects (Figure 1B). Furthermore, when we examined pre-vaccination IL-7Rα expression on ICOS^+^ cT_FH_ cells in the two subgroups of HIV patients, Group A HIV patients exhibited higher than normal expression (Figure 2D). It would therefore appear that we have identified two characteristics of the IL-7Rα signaling pathway in ICOS^+^ cT_FH_ cells that may affect PcP-specific IgG2 antibody responses in HIV patients. Firstly, a decline in IL-7Rα expression on ICOS^+^ cT_FH_ cells between D0 and D7, which may reflect downregulation of IL-7Rα expression consequent upon IL-7Rα signaling (22) and secondly, “supranormal” IL-7Rα expression on ICOS^+^ cT_FH_ cells before vaccination. An explanation for the latter finding is not obvious because of a paucity of information on IL-7Rα expression on ICOS^+^ cT_FH_ cells in HIV patients. However, CD4^+^ T cells expressing high amounts of IL-7Rα are generated in the inflammatory environment of rheumatoid synovitis and, notably, exhibit a much higher proliferative capacity than IL-7Rα^dim^ CD4^+^ T cells (27). It is possible that an analogous process occurs in lymphoid tissue of some HIV patients. The lack of decline of IL-7Rα expression on ICOS^+^ cT_FH_ cells after vaccination in Group B HIV patients, compared with Group A patients and HIV seronegative subjects (Figure 2A), may reflect persistence of impaired IL-7Rα signaling in CD4^+^ T cells observed in some ART-treated HIV patients (25, 26). Our observation that HIV patients exhibiting the greatest decline in IL-7Rα expression had higher current and nadir CD4^+^ T cell counts provides support for this proposition. As Group B HIV patients and HIV seronegative subjects had similar levels of IL-7Rα expression on ICOS^+^ cT_FH_ cells at D0, we propose a model, based on findings reported in Figure 2D, whereby IL-7Rα expression on ICOS^+^ cT_FH_ cells reflects abnormally high IL-7Rα expression or IL-7Rα dysfunction (Supplementary Figure 3) related to persistent inflammation in HIV patients receiving ART. Clarification of this clearly complex situation might be obtained by examining IL-7Rα signaling directly by, for example, assessing intracellular STAT5 phosphorylation.

Analyses of PcP-specific IgG1^+^ and IgG2^+^ ASCs at D7 also provided evidence that IgG2 antibody responses against PcPs were associated with IL-7Rα expression on ICOS^+^ cT_FH_ cells in Group A HIV patients (Figure 6), as well as providing evidence of differences between Group A and Group B patients (Figure 3). One other finding of interest was that ICOS^−^ cT_FH_ cells exhibited increased IL-7Rα expression at D28 in HIV seronegative subjects only, providing evidence in support of the proposition that these cells represent memory cT_FH_ cells (15, 16).

Our findings provide further evidence that at least part of the IgG2 antibody response against the PcP serotypes examined here is generated through a follicular immune response, and possibly a GC reaction given that IgG2 antibody production has been associated with a greater degree of both VDJ gene mutation and SHM wthin immunoglobulin genes compared with IgG3 and IgG1 antibodies (10, 12), and results in expansion of ICOS^+^ cT_FH_ cells (14). As PcPs are TI-2 antigens, this might arise through activation of PcP-specific IgM^+^ MBCs by neutrophils or monocytes exposed to vaccine antigens and the subsequent entry of these cells into follicular and possibly GC reactions (13, 28), where B cell differentiation is regulated by T_FH_ cells. Circulating PcP-specific IgM^+^ MBCs were present before vaccination in the majority of participants in this study (14).

Our study has some limitations, which should be considered when interpreting the findings. The number of study subjects was small, especially amongst the two subgroups of HIV patients, consequent upon a protocol requirement to enroll participants who had not previously received pneumococcal vaccination. Also, there was a sex imbalance between HIV patients and HIV seronegative subjects but we did not observe differences in pneumococcal antibody responses related to sex (data not shown). We did not examine antibody responses after vaccination with PCV13, the use of which is currently standard of care but was not when this study was undertaken. Furthermore, we did not assess plasma IL-7 levels, which inversely correlate with total CD4^+^ T cell counts in HIV patients.

In summary, we provide evidence that production of IgG2 antibodies to PcPs following vaccination of ART-treated HIV patients with PPV23 may be associated with IL-7Rα signaling in ICOS^+^ cT_FH_ cells. Furthermore, we suggest that this finding provides evidence that follicular immune responses contribute to the maturation of antibody responses against PcPs to include IgG2 as well as IgM antibodies. We also suggest that future studies of immune correlates of IgG antibody production after vaccination with conjugated or unconjugated PcPs in HIV patients, and other subjects at risk of pneumococcal disease, might include assessment of IL-7Rα signaling within cT_FH_ cells as a measure of vaccine efficacy and to elucidate regulatory mechanisms that could be modulated to enhance antibody production.

## Ethics Statement

Informed written consent was obtained from all participants and the study was approved by the Human Research Ethics Committees of Royal Perth Hospital, Perth, Australia (2011/027), the University of Western Australia, Perth, Australia (RA/4/1/4871) and Prince of Wales Hospital, Sydney, Australia (11/020).

## Author Contributions

LNA conducted the experiments, analyzed and interpreted the data, wrote and revised the manuscript. SF contributed to the method development and revised the manuscript. KC and L-AK conducted the assay for pneumococcal specific IgG1 and IgG2 antibodies and revised the manuscript. MH and JP assisted in patient recruitment and revised the manuscript. MAF conceptualized the idea, interpreted the data, wrote and revised the manuscript.

### Conflict of Interest Statement

The authors declare that the research was conducted in the absence of any commercial or financial relationships that could be construed as a potential conflict of interest.
