# Supplementary material for: Production of IgG2 Antibodies to Pneumococcal Polysaccharides After Vaccination of Treated HIV Patients May Be Augmented by IL-7Rα Signaling in ICOS+ Circulating T Follicular-Helper Cells
Source: Front Immunol. 2019 Apr 24;10:839. doi: 10.3389/fimmu.2019.00839 (PMC6491457; doi:10.3389/fimmu.2019.00839)
Supplement: Supplementary file 1 [file Table_1.pdf]

Supplementary Table 1. Characteristics of HIV patients and HIV seronegative subjects

Characteristic	HIV Group A	HIV Group B	HIV seronegative subjects
**No. Subjects**	15	10	20
**Age (years)**	56[a]<br>(40 – 73)	49<br>(34 - 63)	52.5<br>(28 – 76)
**Sex, male/female**	15/0[b]	10/0	12/8
**$CD4^+$ T cell count (cells/μL)**	700[c]<br>(340 – 1222)	770<br>(310 – 2058)	985<br>(580 – 1500)
**$CD8^+$ T cell count (cells/μL)**	840[d]<br>(308 – 1836)	1010<br>(564 – 1505)	493<br>(162 – 810)
**Nadir $CD4^+$ T cell count (cells/μL)**	259<br>(24 – 589)	164<br>(16 – 624)	N/A
**B cells (% of lymphocytes)**	9[e]<br>(4 – 21)	12<br>(7 – 22)	15<br>(6 – 25)
**HIV-1 viral load (copies/mL)**	<40	<40	N/A
**Years on ART**	8.45<br>(2 - 21.7)	9.80<br>(1.2 – 20.2)	N/A

[a] Data presented as median (range)

[b] Gender significantly different between HIV Group A (p = 0.006) and HIV Group B ($p = 0.03$) compared to HIV seronegative subjects (Fisher's exact test).

[c] $CD4^+$ T cell counts significantly different between HIV Group A and HIV seronegative subjects ($p = 0.0009$; Mann-Whitney t-test).

[d] $CD8^+$ T cell counts significantly different between HIV Group A ($p = 0.003$) and HIV Group B ($p = 0.0009$) compared to HIV seronegative subjects (Mann-Whitney t-test).

[e] B cells significantly different between HIV Group A and HIV seronegative subjects ($p = 0.007$; Mann-Whitney t-test).
