# Supplementary material for: Production of IgG2 Antibodies to Pneumococcal Polysaccharides After Vaccination of Treated HIV Patients May Be Augmented by IL-7Rα Signaling in ICOS+ Circulating T Follicular-Helper Cells
Source: Front Immunol. 2019 Apr 24;10:839. doi: 10.3389/fimmu.2019.00839 (PMC6491457; doi:10.3389/fimmu.2019.00839)
Supplement: Supplementary file 2 [file Presentation_1.pdf]

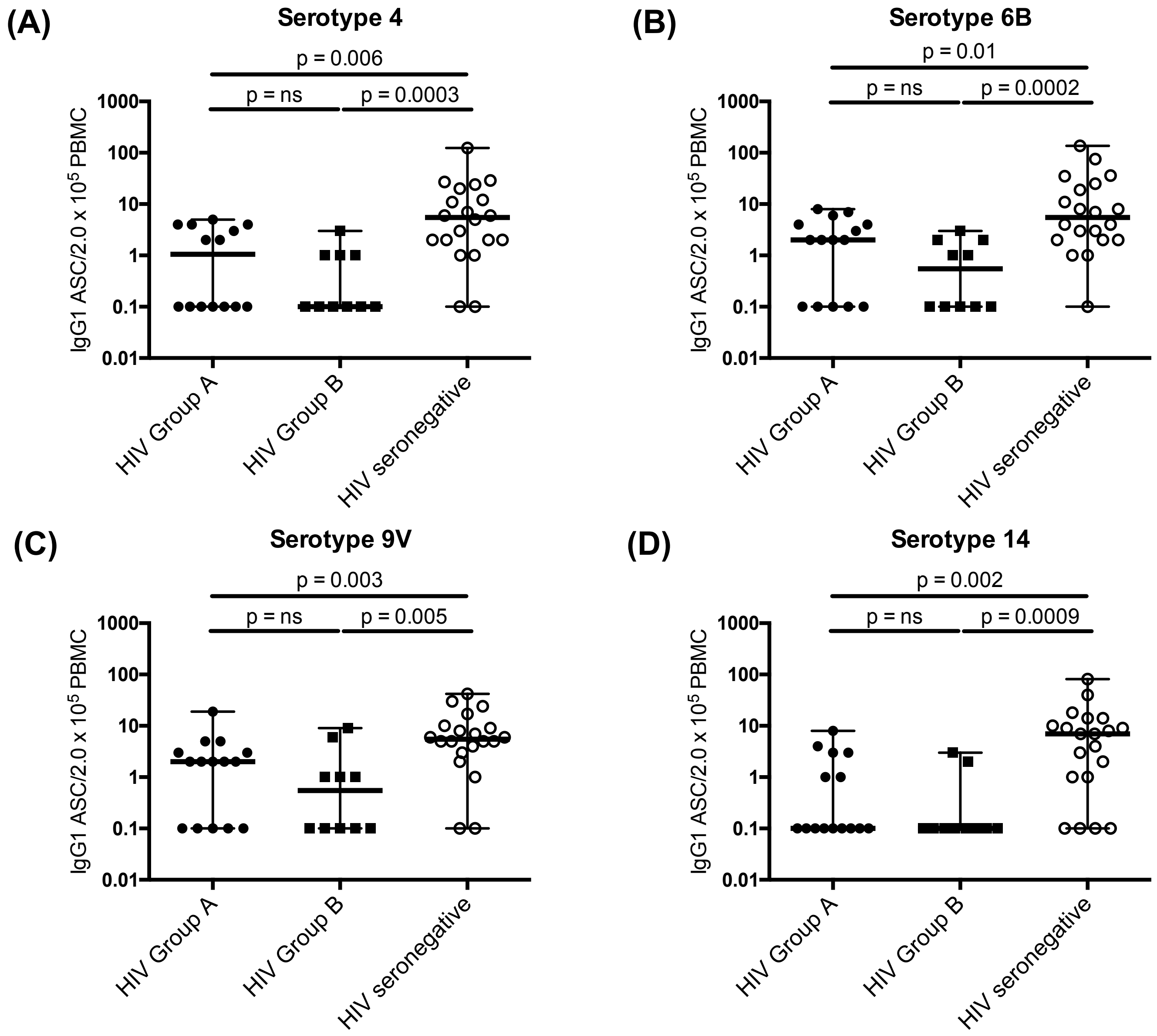

Supplementary Figure 1. **PcP serotype specific IgG1⁺ ASC in Group A HIV patients, Group B HIV patients and HIV seronegative subjects at D7 post-vaccination with PPV23.** (**A**) PcP serotype 4. (**B**) PcP serotype 6B. (**C**) PcP serotype 9V. (**D**) PcP serotype 14. Differences between groups were assessed using Mann-Whitney test. n.s., not significant and $p<0.05$, significant. $IgG1^{+}$ ASC values of 0 were given a value of 0.1 for analysis of data on a log scale.

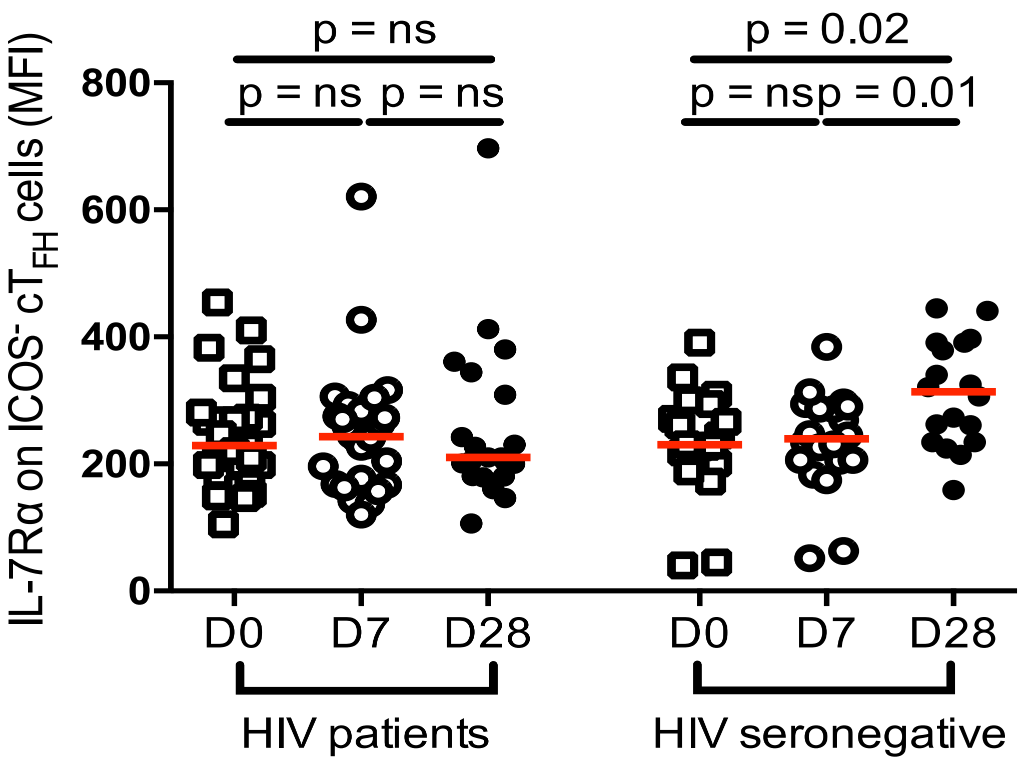

Supplementary Figure 2. Comparison of IL-7Rα expression on ICOS^{-} cT$_{FH}$ cells of HIV patients and HIV seronegative subjects at D0, D7 and D28 after PPV23 vaccination. Differences between time-points were assessed using Wilcoxon signed-rank tests. n.s., not significant and $p<0.05$, significant.

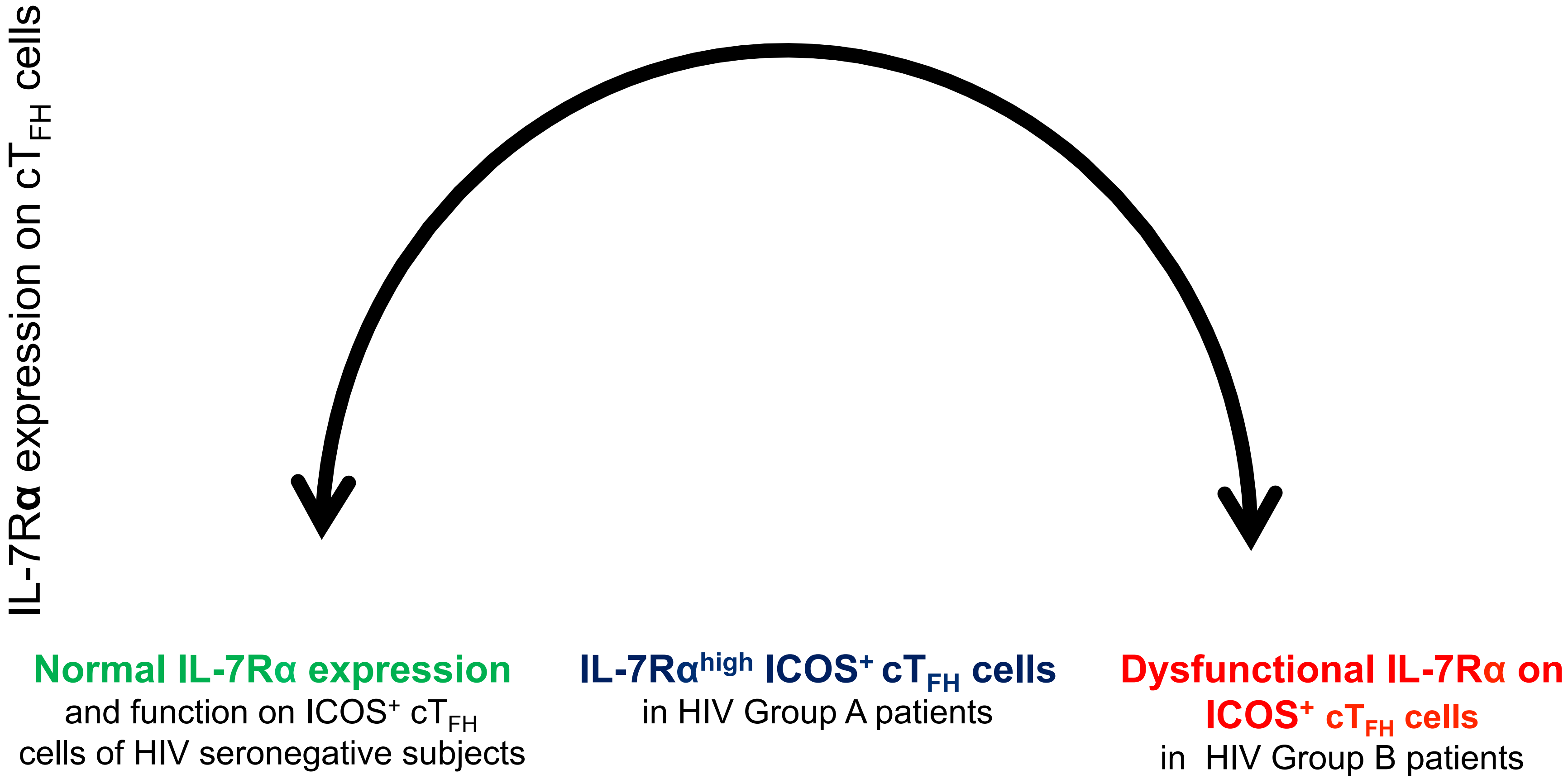

Supplementary Figure 3. Diagrammatic representation of the proposed relationship between level of IL-7Rα expression on cT$_{FH}$ cells and HIV-related abnormalities of ICOS^{+} cT$_{FH}$ cell immunophenotype or IL-7Rα function.
